# Predictors of Treatment Adherence in Kidney Transplant Patients: A Systematic Review of the Literature

**DOI:** 10.3390/jcm14051622

**Published:** 2025-02-27

**Authors:** Edoardo Melilli, María Isabel Díaz, Mar Gomis-Pastor, Esther González, Alex Gutierrez-Dalmau, Enriqueta Isabel Nuño, Ana María Pérez, Inmaculada Plasencia, Ana Sangrador, Esther Lázaro, Nuria Montero, Cristina Soria

**Affiliations:** 1 Nephrology Department, Bellvitge University Hospital, 08907 Barcelona, Spain; n.montero@bellvitgehospital.cat; 2 Bellvitge University Hospital, 08907 Barcelona, Spain; midiaz@bellvitgehospital.cat; 3 De la Santa Creu i Sant Pau Hospital, 08041 Barcelona, Spain; mgomis@santpau.cat; 4 Department of Nephrology, October 12 University Hospital, 28026 Madrid, Spain; montego_12@yahoo.es (E.G.); enriquetaisabel.nuno@salud.madrid.org (E.I.N.); 5 Nephrology Service, Miguel Servet University Hospital, 50009 Zaragoza, Spain; alexgutierrezd@gmail.com; 6 Nephrology Service, Barcelona Clinic Hospital, 08036 Barcelona, Spain; aperezo@clinic.cat; 7 Pharmaceutical Service, The Virgin of Candelaria University Hospital, 38010 Tenerife, Spain; iplagar@gobiernodecanarias.org; 8 Pharmaceutical Service, Marqués de Valdecilla University Hospital, 39008 Santander, Spain; ana.sangrador@scsalud.es; 9 ProHealth Research Group, Sciences Health Faculty, Valencian International University, 46002 Valencia, Spain; 10 Health Psychology, Suportias, 28806 Madrid, Spain; cristina.soria@suportias.com

**Keywords:** kidney transplantation, immunosuppressive medication, adherence, systematic literature review

## Abstract

**Background:** Kidney transplantation (KTx) is a safe procedure that improves the life expectancy and quality of life of patients requiring it. However, despite the known benefits for patients who receive a kidney transplant, non-adherence to immunosuppressive medication is an unsolved problem, reflected mainly by graft rejection. **Objective:** The aim of this study is to systematically review the existing literature on adherence factors to medication after renal transplantation. **Methods:** A systematic literature review of studies published since 2010 was conducted in three databases. Records for the search were limited to publications from 2010 to 2024, available in full-text. The search was carried out in July 2024. In total, 2632 abstracts were downloaded from the different databases. Inclusion criteria were papers of any type (quantitative or qualitative) whose objective was the identification of predictors of adherence for patients who were prescribed immunosuppressive medication after kidney transplantation. **Results:** The predictors of adherence to treatment found in the systematic review were grouped into the following categories of the World Health Organization classification: socio-economic factors, factors related to the treatment/therapy, patient-related factors, disease-related factors, and health care system factors. Most of the studies were excluded, and in the end, 30 were included in the final analysis. According to these studies, a set of strong predictors was identified, but discrepancies among the variables of gender in young patients, pre-emptive transplantation, and the time of the transplantation were detected. **Conclusions:** In this study, we identified specific predictors and directions for the association of those predictors with adherence to immunosuppressive medication for patients after KTx. Further research should consider conducting reviews for different patient sub-groups on medication adherence and the development and validation of a screening instrument for adherence/non-adherence factors that clinicians could use as a detection tool for subjects at risk of low adherence.

## 1. Introduction

Kidney transplantation (KTx) stands as the preferential treatment for end-of-stage kidney disease (ESRD) [1]. KTx is a safe procedure that improves the life expectancy and quality of life of patients requiring such therapy. The number of patients requiring a kidney transplant is growing, both in Europe and in the United States [2,3]. Deceased donor kidney transplantation (DDRTx) is more frequent than from a living donor [2] despite the known benefits for patients who receive a kidney transplant from a living donor (LDRTx). Generally, an organ from a living donor is generally healthier and more resistant to the occurrence and extension of the subsequent ischemia, leading to better graft survival outcomes compared to DDRTx. Moreover, LDRTx have a lower incidence of delayed graft function, thus reducing the period of hospitalization and the need for kidney replacement therapy after transplantation [4].

Graft rejection is a major issue after KTx. Non-adherence to immunosuppressive medication is an important factor for graft rejection and loss after a successful KTx [5,6,7,8,9]. Non-adherence can be highly prevalent in kidney transplant recipients, reaching rates of up to 78%, with a high variability in the results of the studies [5,10,11,12,13,14,15]. A review of recent systematic literature [16] revealed a lack of uniformity in the definition of non-adherence, which explains the wide range of non-adherence prevalence. It is, then, very important to understand the factors that promote better adherence among patients after undergoing KTx [17]. Non-compliance, defined as the voluntary behavior of not following the medication plan recommended for the immunosuppressive medication (contrary to non-adherence, which is not necessarily a voluntary act), has been shown to have similar negative effects for patients, showing that non-compliant patients have higher rates of graft rejection and lower survival rates [18,19].

The World Health Organization (WHO) has provided a classification of factors that have an influence on adherence in general [20]. These include health system factors, socioeconomic factors, factors related to the treatment/therapy, patient-related factors, and disease-related factors. The literature has identified factors that are specifically associated with adherence for patients after KTx. Non-adherence of these patients has, in particular, been associated with a heterogeneous number of factors, including mental health problems, such as anxiety [6,7,12] or depressive symptoms [6,7,11,12,21,22], poor quality of life [10,13,23], beliefs regarding IM [10,12,15,22,24,25], or social support [25]. The perceived susceptibility to rejection and perceived benefits of adherence to treatment have proven to be predictors of adherence rates [26].

The aim of this study is to systematically review the existing literature on adherence factors for medication after renal transplantation based on the WHO classification for adherence factors.

## 2. Materials and Methods

This systematic literature review followed the Preferred Reporting Items for Systematic Reviews and Meta-Analyses (PRISMA) statement [27]. The review protocol was not registered. The framework of this systematic review, according to PIO [28], was as follows: population—people with KTx; intervention—factors of adherence; outcomes—adherence and non-adherence to the treatment recommended by a clinician after KTx.

### 2.1. Search Strategy

A search was conducted in three main databases—Web of Science (WOS), Cochrane Database of Randomized Clinical Trials, and PROQUEST—for this systematic literature review. Other sources (Google Scholar) were also consulted to complete the search, with papers that were not identified in the main databases consulted but that were known to be relevant by the authors of this paper.

To ensure a systematic and comprehensive bibliographic search, key terms were identified through a preliminary review of relevant literature, considering synonyms, linguistic variations, and discipline-specific terminology. Controlled vocabularies like MeSH were consulted for standardized terms. The terms were categorized based on study concepts (population and outcomes). Expert consultation further ensured the terms’ precision and comprehensiveness, supporting replicability and minimizing the risk of missing relevant studies. The PICO method was applied to the structure and combination of keywords regarding KTx (which included “Kidney transplant*” and “renal transplant*”), as well as keywords for adherence (“adherence”, “compliance”). The search equations are included in Table A1.

### 2.2. Eligibility Criteria

Records for the search were limited to publications from 2010 to 2024, available in full-text, in the main databases. The bibliographic search was carried out in July 2024. In addition to the search for articles in English, Spanish language was an additional requirement for records sought in Google Scholar due to the study’s interest in collecting representative predictors that reflect both international and regional realities. The main inclusion criteria were quantitative and qualitative studies evaluating adherence of patients after KTx and identifying adherence or non-adherence factors associated with KTx after transplantation. Exclusion criteria were systematic reviews, patients under 18 years of age, and studies that did not show reliable results (were unfinished or presented unreliable data to draw conclusions, such as case reports).

### 2.3. Study Selection and Data Extraction

After completing the search in each database, all references were imported into Zotero 6.0.22, the software program used for reference management in which the selection of the studies was conducted. The selection of the studies included the screening of all titles and abstracts identified in the database searches in the initial stage and of full texts in the second stage, conducting a forward and backward search. The search and study selection were conducted in July 2024 by two researchers working independently from each other (LE, SC). Any doubts or disagreements arising between the two researchers were discussed with a third researcher (EM). The extraction tool was designed based on recommendations from the Cochrane handbook and adapted to the specificities of this review. The methodology followed for data extraction was reviewed and approved by all authors. It was not necessary to contact any of the authors of the papers included in this review for completion of any missing relevant information from the articles.

### 2.4. Risk of Bias and Quality Assessment of Records

The risk of bias assessment was carried out by two of the co-authors, who discussed the results of the analysis in case of discrepancy until agreement. The method developed by Parmar et al. [29] was chosen for assessing the risk of bias in the records we included. These authors developed a Bias & Quality Assessment tool to be implemented in systematic literature reviews when the review accepts studies from very different sources and methodologies. The tool includes seven key domains: selection bias, ecological fallacy, confounding bias, reporting bias, time bias, measurement error in the exposure indicator, and measurement error in health outcomes. For each publication, each of the above-mentioned domains was scored as follows, and as suggested by the authors who developed the tool: a score of 1 was given for a low risk of bias study, 2 for a moderate risk, and 3 for a high risk. Then, the overall rating was computed as follows: 1 (strong) was given if none of its domains was rated as weak, 2 (moderate) if up to two domains were rated as weak, or 3 (weak) if three or more domains were rated as weak.

Some of these questions needed to be adapted for this review. For example, for randomized controlled trials, because representability does not apply, selection bias was evaluated by analyzing the appropriateness of the sampling methods (e.g., the study shows strong sampling methods reported, large enough sample sizes, blinding, appropriate randomization methods, etc.). Regarding confounding bias assessment, studies that included control variables in their analyses were considered as stronger in terms of quality. Regarding time bias, the longer the period (in years) between the time frame analyzed and the time of publication, the higher the risk of time bias was considered. A higher risk of measurement error in the exposure variable or in the outcome measurement (adherence or non-adherence to medication) was assumed for self-reported measures of adherence, respectively, especially when the measurement instrument used was not a psychometrically validated scale.

## 3. Results

### 3.1. Study Selection

**Figure 1:**
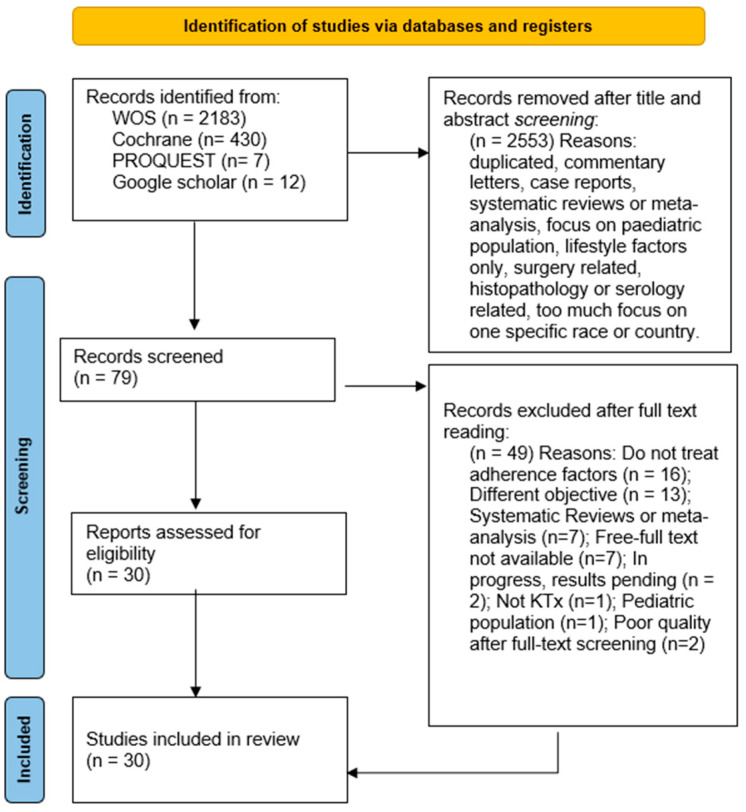
The PRISMA flow diagram.

The search strategy identified 2632 potential studies from WOS (2183), Cochrane Clinical Trials database (430), PROQUEST (7), and Google Scholar (12). After eliminating duplicates and reading the titles and abstracts, 87 articles remained. In the second phase, the complete texts were read, considering the inclusion and exclusion criteria. Finally, 30 articles were selected, from which the information for this systematic review was extracted. Some papers were excluded after performing the risk-of-bias check (*n* = 2). The search and process of exclusion and the selection of the papers are presented in Figure 1 below.

Reference lists of primary research reports were cross-checked in an attempt to identify additional studies.

### 3.2. Risk-of-Bias and Quality Assessment

Each item of the quality and bias scales was rated into one of three categories according to its Risk of Bias as previously described. Among the included studies, there were twenty-one studies rated as strong (having no “weak” ratings) and nine studies considered of moderate quality (with a maximum of one “weak” rating).

After full-text screening and assessment of the quality of the reports and bias assessment, a total of 30 articles, all of them considered to be of moderate or high quality, were kept for the systematic review. Predictors of adherence to immunosuppressive medication as well as for risk of non-adherence to it, were identified from these articles (Table A2).

### 3.3. The Main Results

Table 1 below summarizes the information extracted from the studies ultimately included in this systematic review, including the main and more relevant results for this review (Table 1). Also, the main results are presented in detail in Table 2 with the summary of the 30 studies included in this review (Table 2).

Socio-demographic and economic factors

Socioeconomic factors such as gender, age, education, social support, income, and employment status were found as predictors of non-adherence to IM after KTx. This review found that women are more adherent than men [30] and that aging increases the likelihood for adherence when it is not controlled by gender [25]. Contradictory results of two studies were discovered, one finding young women more adherent than men of the same age in the US and Canada [31], the second one finding young men being more adherent in the UK [32]. In particular, findings show that men are more likely not to adhere to the medication regime recommended by the doctor [33]. Younger individuals, under 50 years of age, were also found to have a higher risk of non-adherence [34,35]. Non-adherence risk was also found to increase for women with high levels of education [36] or for patients with low levels of literacy [22,37]. 

The quality of social support [38] and social interactions appear to be good predictors of adherence, whether it is from social interactions [39], family [40,41], or other patients [41]. A predictor for men seems to be having a good marital relationship [36] and a stable partner [42]. For example, living with parents appeared to be an important predictor for adherence [32], as well as the awareness of the importance of the prescription and recommendations provided by a doctor [43]. 

Economic factors were also found to be good predictors of non-adherence in several of the records included. Specifically, it was found that patients with lower income were at a higher risk of non-adherence [33,43,44], especially if low income was accompanied by high levels of education, as shown in two of the articles included [22,33]. Higher income is also a predictor for non-adherence, but this was found in a study conducted in Brazil, where the lower-income population groups have benefits in terms of better health care coverage and assistance [45]. In relation to demographic variables, rural living and vocational education favored adherence behaviors [35]

Treatment-related factors

In relation to the transplantation process and its follow-up, patients who had at least one previous KTx [46]; had received a transplant from a living organ donor [22]; missed health care appointments [47]; or neglected physical activity recommendations [42]; and those appearing to be concerned about the medication and its complexity, such as the duration of treatment, number of pills, size and taste or side effects, were those most likely not to adhere to the recommended medication [22,33,37,48]. In addition, an increased perception of the symptoms induced by the medical treatment increased the probability of non-adherence, as shown by one of the papers [46]. A lower self-efficacy value regarding medication taking [25,46] and the perception of difficulties in complying with the recommended medication regime, such as challenging nutritional recommendations [44], concomitant medications [30], or complementary medicine [49], were other relevant predictors of the risk of non-adherence found in this category. Finally, in relation to the treatment, the changing of the medication regime to once a day was related to adherence [50].

Patient-related factors

Regarding patient-related factors, being unaware of the complications associated with non-adherence [32,41,43] and other personality traits, such as a reluctance to accept new experiences [11], were found to be good predictors of non-adherence. Other patient-related factors found were the risk of forgetting to take the medication when out of the normal routine [37,51], procrastination [43], low physical function in general [11], depressive syndromes [11,33,34,43], anxiety or emotional distress [22], and a lower QoL in general [34]. Better scores in regard to quality of life matters increase the probability of adherence according to several authors [30,36,39,52]. The ability of patients to self-manage their own health and health care [32], illness acceptance [35], and ability to solve daily-life problems [53] is another example of patient-related factors. 

A consistent finding in two articles was that the self-perception of the severity of the disease when not taking the medication as prescribed [54] or the greater effects of kidney malfunction as perceived by the patient [39] increase the probability of adherence to medication for patients after KTx. A lack of knowledge regarding the utility of the medication prescribed [30], decision making processes comparing cost evaluation against perceived benefits [22], or confusion as to when or which medication to take [30] were also predictors identified in this review.

Disease-related factors

Concerning disease-related predictors, the best predictors of non-adherence were previous dialysis treatment [32,46], except in one study [26]. Other predictors of non-adherence were a greater amount of time since KTx [26,33,38,55] and having other health problems like comorbidities [22,32], cognition problems such as mild cognitive impairment [56], autoimmune nephritis [22], and serious infections after KTx [26].

Health care system factors

In relation to health care system-related factors, satisfaction with the doctor [32] as well as with the condition and resources offered by the facility or clinic where the consultation took place, such as the number of hospital beds or the structure of the waiting room [42], were important predictors found for adherence to the medication of patients after KTx. The absence of health care personnel for consultation in the case of forgetting to take the medication as prescribed [46] and the problem of pharmacies not supplying medications on time [43] were the main predictors of the non-adherence found.

**Table 1:** Conceptual framework of predictors of adherence to medication for KTx patients.

Socio-Demographic and Economic Factors	Related to the Treatment	Related to the Patient	Related to the Disease	Health Care System
Gender:	Type of transplant and characteristics:	Perception of treatment:	↓ More time as of the transplant [26,33,52,55,56]	Satisfaction:
↑ Women [30,33,46]	↓ Living organ donor [22]	↑ Convenience of treatment [44,50]	Other diagnosis:	↑ with the doctor [32]
Age:	↓ Second transplant, at least [46]	↓ Low satisfaction [25,46]	↓ Diagnosis of autoimmune nephritis [22]	↑ with the facilities [57]
↑ Older individuals [25]	↓ The use of complementary medicine [49]	↓ Concerns about medication and its side effects [22,33,37]	↓ Comorbidities [22,32]	↑ with the frequency and duration of the consultation [57]
↓ Below 50 [34,35]	↓ Having had at least one previous KTx [46]	↑ Perception of effects from renal insufficiency [39]	↓ Severe infections [26]	↑ with the explanations given by the doctor [46]
↑↓ Young women compared to young men (≥16 years and <31 years) [32,58]	↓ Complexity of treatment (duration of treatment, number of pills, size and taste, or side effects) [22,33,37,48].	Personality traits, emotions, and perceptions:	↓ Mild cognition problems [56]	Supply problems:
Education:	↓ Appointment non-adherence [47]	↓ Anxiety [11,34,36,37]		↓ Lack of doctors for consultation when having doubts [46]
↓ High education level and being a woman [36]	↑ Medication once a day [50]	↓ Depression [22,41,48]		↓ Lack of medication [37,43]
↓ High level of education at the same time as low income levels [22,33]	Other treatments:	↑ Awareness of the importance of medical recommendations [32,41,43]		
↓ Low alphabetization levels [56]	↓ Other concomitant immune-suppressive medications (it is not clear what the other concomitant medications are) [30]	↑ Ability to self-manage health [32]		
Income:	↑↓ Previous dialysis treatment [26,32,46]	↑ Ability to problem-solve [53]		
↓ Low income [37,43,44]	↑↓ Pre-emptive transplantation [26,32,46]	↓ Difficulties in remembering which medication to take or when [30,37,47,51]		
↑ Rural living [35]		↓ Limited willingness to face new experiences [11]		
Employment status:		↓ Lack of knowledge about the utility of the medication [30]		
↓ Employed, with very little spare time [22,43,51]		↑ Better health-related quality of life [30,36,39,52]		
Social support:		↑ Illness acceptance [35]		
↑ Being married [54], in a good relation with the partner [36] or in a stable relationship [57]				
↑ Social support [36,52] and quality of social interactions [39]				
↑ Family support [40,41]				
↑ Living with parents [32]				
↑ Support from other patients [41]				

↑ predicts adherence; ↓ predicts non-adherence; ↑↓ contradictory results were found.

**Table 2:** Summary of studies: description, objective, and main findings.

Reference, Country, Study Type and Analysis, Sample	Objective	Main and Significant Results Found
Boucquemont et al., 2019 [31] US and Canada Randomized study without intervention adherence follow-up using pillboxes. Ordinal logistic regression N = 136 patients between 11–16 years old vs. 17–24 years old	To determine whether adherence differs by gender and whether gender differences vary by age in adolescent and young adult kidney transplant recipients.	Socio-demographic factors: Gender differences in adherence vary by age. Young women (17–24) show much greater adherence than young men. Patient-related factors: NA. Treatment-related factors: NA. Disease-related factors: NA. Health care system-related factors: NA.
Taber et al., 2017 [47] US Longitudinal cohort study N = 3656 complete records	To determine if appointment non-adherence was correlated with medication non-adherence and a predictor of graft outcomes.	Socio-demographic factors: NA. Patient-related factors: NA. Treatment-related factors: Failure to keep the appointment and medication were highly correlated, and both were important independent predictors of outcome. There is a strong correlation between appointment-keeping and medication non-adherence in kidney transplant recipients, and appointment non-adherence is a strong predictor of graft loss and death, even after accounting for non-adherence to medication and acute rejection. Failure to comply with medications and appointment visits was associated with a 4-fold increase in the risk of graft loss. Disease-related factors: NA. Health care system-related factors: NA.
Hamilton et al., 2018 [32] UK Online cross-sectional survey Multivariate linear Regression N = 976 young adults (16–31 years old). Final sample = 432	To test the hypothesis of whether well-being and medication adherence are associated with psychosocial factors.	Socio-demographic factors: Greater adherence to medication was associated with living with parents, 16–21 years of age, and the male sex. Patient-related factors: Greater adherence to medication was associated with conscientiousness as a personality trait (this dimension is based on self-control, not only of impulses, but also in the planning, organization, and execution of tasks. Treatment-related factors: Lower adherence was associated with dialysis. Dialysis treatment was associated with poorer well-being and less medication adherence. Disease-related factors: Lower adherence was associated with comorbidity (undetermined) and dialysis. Dialysis treatment was associated with poorer well-being and reduced medication adherence. Health care system-related factors: Greater adherence to medication was associated with living with parents and satisfaction with access to the doctor.
Weng et al., 2017 [33] Taiwan Cross-sectional, correlational study, multiple regression. Use of structured questionnaires N = 145	To determine the factors related to immune-suppressant therapy adherence in kidney transplant recipients in Taiwan.	Socio-demographic factors: Factors related to reduced adherence were male sex; low income + secondary education, and low income with college education. Patient-related factors: Factors related to less adherence scored higher on the medication concerns sub-scale. Treatment-related factors: Factors related to reduced adherence increased with the number of years after kidney transplantation. Disease-related factors: NA. Health care system-related factors: NA.
Cossart et al., 2019 [51] Australia Cross-sectional, correlational study N = 161	To investigate the prevalence of non-adherence and barriers to adherence with immune-suppressive medications in an adult renal transplant cohort.	Socio-demographic factors: NA. Patient-related factors: Non-adherent patients were less able to tell if their medications were helping them and were likely to forget or skip medication doses when they were outside of their normal daily routine. They showed less understanding about their kidney graft. They tended to skip or delay the dose. Non-compliant patients were more likely to forget or skip doses when out of the normal routine or financially challenged. They were more likely to be employed as they were generally more time-constrained. Adherence was not associated with an individual’s concerns, needs and beliefs about their medication, or perceived locus of control. Treatment-related factors: NA. Disease-related factors: NA. Health care system-related factors: NA.
Paterson et al., 2018 [53] Canada Correlational cross-sectional study, structural equations. Self-report measures, Medication Possession Index, and blood level of immuno-suppressants. N = 211	To gain a better understanding of the predictors of adherence in renal transplant recipients.	Socio-demographic factors: NA. Patient-related factors: Predictors of Self-Reported Medication Adherence—the model highlights the importance of everyday problem-solving (EPS) ability. Both self-efficacy and daily problem solving (PSS) directly and positively predict self-reported adherence. Neither depressive symptoms nor neurocognitive abilities are directly associated with adherence. Treatment-related factors: NA. Disease-related factors: NA. Health care system-related factors: NA.
Griva et al., 2012 [22] Singapore Cross-sectional study with survey N = 152	To compare the rates and determinants of unintentional, intentional, and overall self-reported non-adherence.	Socio-demographic factors: Participants who reported involuntary non-compliance were more likely to have a formal job. Participants who were classified as intentionally non-adherent had a higher education. Patient-related factors: Overall non-compliance was predicted by a deliberate decision-making process that involved weighing the costs of using immune-suppressive drugs against the perceived benefits. Participants who reported involuntary non-compliance had higher levels of anxiety, symptoms, and general emotional distress compared with those who were classified as adherent. Participants who were classified as intentionally non-adherent expressed more concerns regarding their medication compared to those who were classified as adherent. Participants who were classified as non-adherent generally had more concerns about their medications and assessed the benefits of the over-medication side effects more negatively compared with those who were classified as adherent. Treatment-related factors: Unintentional non-adherence was predicted by primary diagnosis of autoimmune nephritis or a recipient of a living donor kidney transplant. Intentional non-adherence was predicted by the side effects of their medications. Disease-related factors: Intentional non-adherence was predicted by a comorbidity burden. Participants who reported involuntary non-compliance had autoimmune nephritis as the cause of their end-stage renal disease. Health care system-related factors: NA.
Kung et al., 2017 [26] China Cross-sectional study with survey N = 122	To understand the influence of the personal characteristics and health-related beliefs of patients on adherence to treatment with immunosuppressive medication based on the Health Belief Model.	Socio-demographic factors: NA. Patient-related factors: NA. Treatment-related factors: Adherence to treatment with immunosuppressive medication was significantly and negatively correlated with time since renal transplantation, suggesting that compliance rates decreased over time. Participants who had experienced severe to extremely severe infections had lower rates of adherence to treatment with immune-suppressive medication than those who had experienced minor infections. Participants who experienced a greater number of drug-induced symptoms had lower rates of treatment adherence. Participants who had received dialysis, those who had experienced rejection, or those who had a relatively stronger susceptibility to rejection expressed strong motivation for adherence to medication treatment because they were afraid of experiencing rejection, which would lead to kidney failure, dialysis dependence, and poor quality of life. Disease-related factors: NA. Health care system-related factors: NA.
Kobayashi et al., 2020 [46] Japan Cross-sectional study with survey N = 219	To clarify the prevalence and risk factors of patient non-adherence after kidney transplantation in Japan.	Socio-demographic factors: Factor associated with non-adherence was male sex. Patient-related factors: Factor associated with non-adherence was low self-efficacy in relation to medication. Treatment-related factors: Factor associated with non-adherence: short dialysis period before transplantation. Disease-related factors: NA. Health care system-related factors: Factors associated with non-adherence were lack of satisfaction with the explanation of the immune-suppressive medication and lack of medical personnel to consult in case of forgetting to take medication.
Xia et al., 2019 [54] China Cross-sectional study with survey N = 208	To examine the beliefs in IM and IM adherence in Chinese renal transplant recipients.	Socio-demographic factors: After controlling other factors, the following were significantly associated with adherence. Marital status. Patient-related factors: High levels of perceived severity of not taking medication. Treatment-related factors: NA. Disease-related factors: NA. Health care system-related factors: NA.
Zhao et al., 2018 [52] China Descriptive, correlational, and cross-sectional design N = 253 recipients of KTx	This study assessed QoL, adherence behavior, social support, and their relationships among renal transplant recipients.	Socio-demographic factors: Increased social support and strong positive relationships were found among most areas of quality of life, adherence behavior, and levels of social support. Social support was the most significant factor influencing adherence behavior and quality of life. Patient-related factors: NA. Treatment-related factors: Greater adherence was associated with a shorter amount of time since transplant (recipients who were between 0.5 and 1 years after transplantation) Disease-related factors: NA. Health care system-related factors: NA.
Chironda et al., 2019 [41] South Africa Phenomenological qualitative design through semi-structured interviews N = 12	To explore the motivators of adherence to integrated management among patients with CKD in South Africa.	Socio-demographic factors: NA. Patient-related factors: Five main themes emerged from the experiences cited by CKD patients as motivators toward adherence: Family support; anxiety about eligibility for kidney transplantation; support from other CKD patients. Awareness of complications associated with non-adherence to integrated management. Treatment-related factors: NA. Disease-related factors: NA. Health care system-related factors: NA.
Zhao et al., 2017 [38] China Cross-sectional study with survey N = 250	To investigate the status of adherence to follow-up and QoL and associated factors among kidney transplantation recipients in China.	Socio-demographic factors: NA. Patient-related factors: NA. Treatment-related factors: Adherence decreases with more time after transplantation. Disease-related factors: NA. Health care system-related factors: NA.
Cheung et al., 2021 [40] China Cross-sectional study with survey N= 210	To determine the various dimensions of IM non-adherence in KTRs during the COVID-19 pandemic.	Socio-demographic factors: Impact of the COVID pandemic on adherence was not significant. However, family support may have a positive influence on adherence during the pandemic. Patient-related factors: NA. Treatment-related factors: NA. Disease-related factors: NA. Health care system-related factors: NA.
Neubert et al., 2021 [36] Cross-sectional study with survey N = 56 couples with a KTx recipient	Identifying factors that influence adherence.	Socio-demographic factors: For male patients, adherence was significantly correlated with perceived social support and marital quality. Patient-related factors: For male patients, adherence was significantly correlated with mental and physical quality of life. For male kidney transplant recipients, significant predictors of adherence emerged, such as social support, quality of personal relationships, and quality of life, while for female kidney transplant recipients, it was found that mental quality of life and educational level influenced adherence. Treatment-related factors: NA. Disease-related factors: NA. Health care system-related factors: NA.
Costa-Requena et al., 2017 [55] Spain Longitudinal study (24 months) N = 73	To longitudinally assess the adherence to treatment after kidney transplant and compare the amount of information about the treatment received at 1 month and 18 months post-transplantation and its influence on adherence to treatment.	Socio-demographic factors: NA. Patient-related factors: NA. Treatment-related factors: The longer the period of time since transplantation increased non-adherence to treatment. Disease-related factors: NA. Health care system-related factors: NA.
Sanders-Pinheiro et al., 2021) [42] Brazil Observational, cross-sectional, multi-center study including 20 Brazilian KTx centers N = 1105	Immuno-suppressive non-adherence is a risk factor for worse outcomes after kidney transplantation (KT).	Socio-demographic factors: Some factors were independently associated with non-adherence. Patient level: having a stable partner and non-compliance with physical activity recommendations. Patient-related factors: Some factors were positively associated with lack of adherence: appointment failure and non-compliance with physical activity recommendations. Treatment-related factors: NA. Disease-related factors: NA. Health care system-related factors: Some factors were independently associated with non-adherence: satisfaction with the structure of the waiting room, consultation >30 min, adequacy consultation frequency, and centers with >500 beds.
Villeneuve et al., 2021 [34] France Longitudinal study of adherence scales with two cohorts N = 345 + 367	Adherence is a dynamic phenomenon and a critical determinant of transplant patients’ outcome. The objective of this longitudinal study was to explore adherence in kidney transplant patients followed-up during up to a three-year period transplantation.	Socio-demographic factors: Being of a younger age (<50 years) acts as a predictor of belonging to the non-adherent class. Patient-related factors: In the non-adherent class: more depressive syndromes and lower quality of life. Treatment-related factors: NA. Disease-related factors: NA. Health care system-related factors: NA.
Markell et al., 2021 [49] US Cross-sectional study with survey N = 51	To examine the relationship between self-reported use of CAM, attitudes toward care and adherence to medical therapy in a population of inner-city kidney transplant recipients.	Socio-demographic factors: NA. Patient-related factors: NA. Treatment-related factors: Patients using complementary medicine were more likely to be non-compliant with non-immunosuppressive drugs. Disease-related factors: NA. Health care system-related factors: NA.
Patzer et al., 2021 [56] US Prospective study with survey N = 99	To evaluate the prevalence of understanding the medication and non-adherence of entire drug regimens among kidney transplantation (KT) recipients and to examine associations of these exposures with clinical outcomes.	Socio-demographic factors: Lack of adherence was associated with Limited Literacy (assessed using REALM— a brief screening instrument used to assess an adult patient’s ability to read common medical words). Patient-related factors: NA. Treatment-related factors: Lack of adherence was associated with fewer months since the transplant. Disease-related factors: Lack of adherence was associated with mild cognitive impairment. Health care system-related factors: NA.
Oh et al., 2020 [50] Prospective trial South Korea N = 160	To investigate medication adherence of simplified once-daily immune-suppressive regimen consisting of extended-release tacrolimus, sirolimus, and corticosteroids along with the efficacy and safety of this regimen.	Socio-demographic factors: NA. Patient-related factors: NA. Treatment-related factors: Medication adherence improved after conversion to the once daily immunosuppressive regimen, without additional risks of efficacy failure or adverse events. Disease-related factors: NA. Health care system-related factors: NA.
Weng et al., 2013 [37] US Cross-sectional study with survey N = 252	To determine the prevalence and correlates of medication non-adherence among kidney transplant recipients.	Socio-demographic factors: NA. Patient-related factors: NA. Treatment-related factors: Seven of the thirteen individual items on the Therapeutic Barriers Scale-ITBS were significantly associated with adherence: I go out of town. Feel depressed; difficult to remember; side effects. Don’t have money. Disease-related factors: NA. Health care system-related factors: NA.
Marsicano et al., 2015 [45] Brazil Cross-sectional study with survey N = 100	To study the correlates of immune-suppressive non-adherence in post-kidney transplant patients in the Brazilian health care system.	Socio-demographic factors: Immuno-suppressive non-adherence was associated with higher family income. Patient-related factors: NA. Treatment-related factors: NA. Disease-related factors: NA. Health care system-related factors: NA.
Russell et al., 2013 [25] US Longitudinal correlational study N = 121	This study examined patterns, potential predictors, and outcomes of immune-suppressive medication adherence in a convenience sample of 121 kidney transplant recipients aged 21 years or older from three kidney transplant centers using a theory-based, descriptive, correlational, longitudinal design. Electronic monitoring was conducted for 12 months.	Socio-demographic factors: Advanced age was the only demographic variable associated with medication adherence. Patient-related factors: Medication self-efficacy was associated with non-adherence. Treatment-related factors: NA. Disease-related factors: NA. Health care system-related factors: NA.
Ganjali et al., 2019 [30] Iran Descriptive, cross-sectional study application to test kidney transplant patients N = 244	To determine the risk factors associated with adherence to immunosuppressive regimen and its barriers among kidney transplant (KT) recipients.	Socio-demographic factors: Women were twice as likely to be adherent than men. Patient-related factors: Kidney transplant recipients with a higher quality of life were more likely to continue immune-suppressant medicine. The relevant factors that imply a difficulty in adherence were the concurrent use of many immune-suppressants, lack of knowledge about the usefulness of immunosuppressants, confusion in taking medications, and difficulty remembering the medication that is taken. Treatment-related factors: NA. Disease-related factors: NA. Health care system-related factors: NA.
Israni et al., 2016 [43] US Qualitative study with semi-structured survey N = 16	The aim of this paper is to determine how kidney transplant recipients remember to take their medications and assess their perception and beliefs about adherence to immune-suppressive medications and barriers to medication adherence. In addition, we aim to assess perception and beliefs about the willingness to use a hypothetical mobile phone app to improve adherence.	Socio-demographic factors: NA. Patient-related factors: Reasons for taking immune-suppressive drugs: to avoid dialysis; because it was prescribed by the doctor; to stay alive; to prevent rejection; becoming adherent due to a death in the family caused by non-adherent behavior. Perceived barriers for taking immune-suppressive drugs: forgetting to bring medication to work; inflexible work hours; procrastinating when taking medication; procrastinating when ordering medication; patients cannot feel the damage they are inflicting on their kidneys due to a shortage of money or being depressed; the pharmacy did not supply the medicine on time; the patient forgot to take it due to distractions; the patient did not want to take medication with alcohol; the patient forgot to renew his prescriptions; patient changed pharmacy (due to insurance coverage); drugs were stolen; The patient forgot to take his medicine when he was away from home; the patient overslept and forgot the afternoon medications; taking medications at a different time than usual because the patient’s minimum levels are controlled in the laboratory. Perceived barriers to keeping appointments: being distracted by children; not having transportation; double booking of medical appointments; disease; reminder too far in advance; falling asleep; forgetting the appointment Treatment-related factors: NA. Disease-related factors: NA. Health care system-related factors: NA.
Gorevski et al., 2013 [11] US Cross-sectional study Logistic regression analyses N = 86	To measure the association of transplant patients’ personality, depression, and quality of life with medication adherence in kidney and liver transplant recipients.	Socio-demographic factors: NA. Patient-related factors: Association between the patient’s lack of adherence and the presence of depression, personality traits characterized by low openness to experience (O), [openness to experience is defined by preference for variety, intellectual curiosity, and independence of judgment compared to low openness, in which one tends to be conventional in behavior, preferring the familiar to the novel (assessed with the NEO-FFI Scale) and poor physical functioning (assessed with the Karnofsky Performance Status Scale) in heart transplant patients. Treatment-related factors: NA. Disease-related factors: NA. Health care system-related factors: NA.
Matos Trevín et al., 2019 [44] Cuba Cross-sectional study with survey N = 75	To characterize, according to levels, the therapeutic adherence of patients with chronic renal failure on dialysis treatment and to describe the factors that condition it.	Socio-demographic factors: NA. Patient-related factors: The perception of treatment as difficult to comply with was a factor described as a condition for poor adherence and the economic possibilities for carrying it out, especially the diet indicated, were selected by all the partially adherent and non-adherent patients as the cause of non-compliance with the indications. Treatment-related factors: Indications that the patients participating considered it more difficult to comply were fluid restriction as the most difficult aspect to comply with in their medical treatment, followed by the indicated diet. Disease-related factors: NA. Health care system-related factors: NA.
Díaz-Soto et al., 2017 [39] Colombia Prospective cross-sectional study N = 75	To analyze the relationship between health-related quality of life factors and adherence to treatment in patients with CKD undergoing renal replacement therapy. The study was analytical, prospective, and cross-sectional.	Socio-demographic factors: NA. Patient-related factors: Adherence to treatment in patients with CKD was associated with some of the factors of health-related quality of life, such as greater effects of renal failure, greater burden of renal disease, better quality of social interaction, greater emotional well-being, greater emotional role, and greater physical component. Treatment-related factors: NA. Disease-related factors: NA. Health care system-related factors: NA.
Zachcial et al., 2022 [35] Poland Cross-sectional study	To evaluate the association of transplant patients’ acceptance of illness, symptoms of anxiety and depression, frailty, and QoL with medication adherence in KT recipients.	Socio-demographic factors: Rural living and vocational education. Patient-related factors: Acceptance of illness. Treatment-related factors: NA. Disease-related factors: NA. Health care system-related factors: NA.

## 4. Discussion

This systematic review is aimed at assessing the evidence available on the predictors of adherence and non-adherence to immune-suppressive medication by patients who underwent KTx. The goal was to identify literature gaps, document the current cutting-edge knowledge, and initiate a discussion regarding the direction in which further research should be focused. To our knowledge, this is one of the few studies that concentrate exclusively on the identification of specific predictors and the association of those predictors with adherence to immune-suppressive medication in patients after KTx.

Our review results indicate that socio-demographic factors, patient-related factors, treatment-related factors, disease-related factors, and health care system-related factors are good categories for establishing the predictors of both adherence and non-adherence to immune-suppressive medication in the targeted patients. This structure was proposed by the WHO, and it is appropriate for categorizing adherence and non-adherence predictors within the context of this review.

In view of the number of abstracts collected by our search strategy, one might think that there is extensive literature on this topic. However, a large number of studies were excluded (N = 2553 excluded abstracts in the abstract screening phase, and 58 excluded records in the full-text screening phase), and the final number of records included (n = 30) represented 1% of all the records found and 32% of the full-text records screened (as stated in the PRISMA flow diagram).

There are several contradictory findings that need to be explored in greater depth by expanding research to further study these matters and increasing sample sizes of similar studies in the future. For example, contradictions were found for gender: some studies found women were more adherent than men [31], and other studies found the opposite situation in favor of men [32]. Differences in adherence results by gender may be explained, in part, by the different methodologies used in the studies analyzed. For example, one study [31] found that young women had higher adherence compared to young men using a randomized study design with no intervention and follow-up with electronic pillboxes. In contrast, another study [32], a cross-sectional survey study in the UK, found that young men showed higher adherence when they lived with their parents and were associated with high levels of awareness about the importance of treatment. These methodological differences could be influencing the findings, as studies using self-reporting tend to reflect perception biases, while those using objective measures, such as the use of electronic pillboxes, may capture more accurate data on actual adherence behavior. Another source of discrepancy is the variability in the contextual factors considered in each study. While some studies examine adherence based on psychosocial factors such as family support and quality of life, others focus on demographic or economic variables. The impact of gender on adherence might be mediated by factors such as education, economic stability, and social support structure. The lack of a homogeneous approach in the selection of variables and assessment methods highlights the need for more standardized studies that would allow for more consistent conclusions about the role of gender in treatment adherence.

Similarly, being on dialysis treatment before the kidney transplant was found to be a predictor for both adherence [26] and non-adherence [32,46]. Another contradiction was found in regard to the amount of time elapsed since the transplantation; some studies found that the more recent the transplantation [56], the higher the risk of non-adherence, and other studies found that longer periods of time since the transplantation are associated with higher probabilities for non-adherence [26,33,38,55]. 

This review represents an improvement over previous systematic reviews carried out, as it focuses exclusively on describing in detail the existing evidence on the factors related to adherence to treatment. This review contributes to the existing literature by applying the WHO framework to deepen the understanding of adherence factors, categorizing them into socioeconomic, treatment-related, patient-related, disease-related, and health care system factors [59,60]. The evidence should be accompanied by quality indicators as a guarantee of the reliability of the conclusions. Possessing structured evidence is crucial to enhancing knowledge about the factors that should be the focus of attention, both for establishing screening programs for patients at risk of non-adherence and for designing personalized intervention strategies focused especially on those modifiable strategies. In this sense, it is important to anticipate those factors that predict good adherence, as well as those predictors of non-adherence. In this way, clinicians can take steps in advance with patients showing a high risk of non-adherence. Therefore, it is necessary for clinicians to rely on validated tools that will help them anticipate situations of risk of non-adherence, thus increasing the chances that patients will adhere to medical prescriptions and show clinical improvement and health-related quality of life. Further research should focus on the development and validation of a suitable screening instrument for adherence/non-adherence factors that will serve clinicians as a detection tool for subjects at risk of low adherence. In addition, this type of screening tool has to be accompanied by specific and validated protocols and guidelines for action once these factors are identified in clinical practice, and they will help in adopting individualized clinical decisions for each patient and their specific environment.

Our paper has several limitations. First, the evidence was mostly based on self-reported measures of adherence and non-adherence and does not allow for a true extrapolation of the results in most studies due to questions either of small sample sizes and/or selection bias. In many studies, there was an under-representation of the population of non-adherent patients merely because of the requirement that the participant had to be able to attend the consultation. This also justifies the fact that many studies had a small sample size, given the difficulties of recruiting patients to participate in the study. Second, contrary to other reviews [16], we did not include papers published before 2010. The reason for this limitation is that most publications were issued after this year and also that most measures for adherence of patients were only validated in this past decade. Moreover, immune-suppressant protocols prior to 2010 were quite different from those today, so we considered that a study focusing on papers as of 2010 would be more accurate. In this sense, the focus of this review is on predictors of adherence, but the existing heterogeneity of adherence measures is an interesting topic to address in future research focused on measures. Third, given that the objective of the review was to determine adherence and non-adherence predictors, in order to fill the gaps left by previous literature, papers of all kinds of methodologies were included. This means that the identification of predictors includes sources that range from purely theoretical works to purely quantitative or qualitative studies, which could lead to certain conflicts and difficulties in aggregating predictors within categories or themes or might even be the reason for the contradictions found. Socio-demographic and economic factors appear to be highly dependent on the type of health care system in which the transplant was performed—for example, US health care systems versus universal health care systems. This aspect should be considered when interpreting the results of this review. Also, restricting the inclusion to English and Spanish studies may have led to the omission of relevant research in other languages, potentially affecting the generalizability of the findings, especially for Asian and European populations. Finally, this review identified predictors of adherence and non-adherence for a specific type of patient, and therefore, it may not be a framework that could be applied to other patient sub-groups. This study includes considerable heterogeneity in study design, populations, and outcome measures; however, the lack of a formal heterogeneity assessment and subgroup analysis limits the interpretation of findings across different contexts. The lack of a standardized definition of adherence and variation in measurement tools limits meaningful comparisons across studies and could be addressed in future research. It would be relevant to conduct a meta-analysis in future studies, as it could provide valuable insights by exploring the magnitude and precision of associations across more homogeneous study designs, populations, and outcome measures. Further research should explore these specific factors. Other suggestions would be to explore the possibility of conducting similar studies on adherence, focusing on the importance of using validated measures in assessing medication adherence. Following guidelines could improve the quality of the literature on this important topic and explain some of the contradictory findings detected in our review [61].

## 5. Conclusions

This review provides a conceptual framework for predictors of adherence and non-adherence of patients who underwent KTx. This framework is based on the WHO proposed categories for medication adherence but provides specific predictors and directions of associations for those predictors of adherence and non-adherence in the targeted patients. This framework is specific to this patient population and cannot be used for other patient populations. Further research should consider conducting reviews on different patient sub-groups regarding medication adherence in order to be able to inform health care institutions and providers of health care services on how to improve adherence to medication regardless of the condition of the patients.

## Data Availability

Data are contained within the article.

## Appendix A

**Table A1:** Search equations.

WOS
[[Kidney transplant* OR renal transplant* (Topic)] AND [adherence OR patient compliance (Topic)]]
**Cochrane**
[[(kidney transplant* OR renal transplant*):ti,ab,kw] AND [(adherence OR patient compliance):ti,ab,kw]]
**Proquest**
[title(kidney transplant* OR renal transplant*) AND title(adherence OR compliance)]

## Appendix B

**Table A2:** Evaluation of the risk of bias and quality assessment of the studies included in the review according to the Parmar et al.’s assessment scale [29].

	Risk of Bias and Quality Assessment Dimensions							Global Score
Reference	SB	EF	CB	IB	TB	EME	EOM	
Boucquemont et al., 2019 [31]	1	1	1	3	1	1	2	2
Taber et al., 2017 [47]	3	2	1	1	1	1	2	2
Hamilton et al., 2018 [32]	2	2	1	1	1	1	1	1
Weng et al., 2017 [33]	3	2	1	1	2	1	1	2
Cossart et al., 2019 [51]	2	2	1	1	1	1	1	1
Paterson et al., 2018 [53]	2	2	1	1	1	1	1	1
Griva et al., 2012 [22]	2	2	1	2	1	1	1	1
Kung et al., 2017 [26]	2	2	1	2	2	2	2	1
Kobayashi et al., 2020 [46]	2	2	1	2	2	2	2	1
Xia et al., 2019 [54]	2	2	1	2	1	2	2	1
Zhao et al., 2018 [52]	2	2	1	2	1	2	2	1
Chironda et al., 2019 [41]	2	2	1	2	1	2	2	1
Zhao et al., 2017 [38]	2	2	1	2	1	2	2	1
Cheung et al., 2021 [40]	2	2	1	2	1	2	2	1
Neubert et al., 2021 [36]	2	2	1	2	1	2	2	1
Costa-Requena et al., 2017 [55]	3	2	2	2	1	2	2	2
Sanders-Pinheiro et al., 2021 [42]	2	2	1	1	1	1	1	1
Villeneuve et al., 2021 [34]	1	1	1	1	1	1	1	1
Markell et al., 2021 [49]	3	2	2	2	2	2	2	2
Patzer et al., 2021 [56]	3	1	1	2	2	2	2	2
Oh et al., 2020 [50]	2	1	1	2	2	2	2	2
Weng et al., 2013 [37]	3	1	1	2	2	2	2	2
Marsicano et al., 2015 [45]	3	1	1	2	2	2	2	2
Russell et al., 2013 [25]	1	1	1	1	2	1	1	1
Ganjali et al., 2019 [30]	2	1	1	2	2	2	2	1
Israni et al., 2016 [43]	2	1	1	2	2	2	2	1
Gorevski et al., 2013 [11]	2	1	1	2	2	2	2	1
Matos Trevín et al., 2019 [44]	2	1	1	2	2	2	2	1
Díaz-Soto et al., 2017 [39]	2	1	1	2	2	2	2	1
Zachcial et al., 2022 [35]	2	1	1	2	2	2	2	1

Abbreviations: SB—selection bias; EF—ecological fallacy; CB—confusion bias; IB—information bias; TB—time bias; EME—measurement error in exposure indicator; EOM—measurement error in health outcome. Punctuations: 1—strong; 2—moderate; 3—weak.
